# Proportion of Immediate Postpartum Anaemia and Associated Factors among Postnatal Mothers in Northwest Ethiopia: A Cross-Sectional Study

**DOI:** 10.1155/2020/8979740

**Published:** 2020-06-16

**Authors:** Asenake Abebaw, Temesgen Worku Gudayu, Bayew Kelkay

**Affiliations:** ^1^Debre Birhan Health Science College, Debre Birhan, Ethiopia; ^2^School of Midwifery, College of Medicine and Health Sciences, University of Gondar, Gondar, Ethiopia

## Abstract

**Background:**

Anaemia is a major global health problem, especially in developing countries. Postpartum anaemia hurts both maternal and newborn baby health. Anaemia in pregnancy is sufficiently emphasized; however, very little attention has been paid to postpartum anaemia in Ethiopia. Therefore, this study aimed to investigate the proportion of immediate postpartum anaemia and associated factors among postpartum mothers in Debre Markos Referral Hospital.

**Methods:**

Institutional-based cross-sectional study was conducted among 424 study participants from August 1^st^ to October 30^th^, 2019. A systematic random sampling technique was employed to select the study participants. Data were collected through both face-to-face interview and maternal chart review by using a pretested questionnaire. Data were cleaned, coded, and entered using Epi Data version 4.6.0.0 and then exported to SPSS version 24 for analysis. First, binary logistic regression was applied to identify candidate variables for multivariable regression. Then, variables at *p* value <0.2 were entered into a multivariable logistic regression to control possible confounders. Finally, variables at *p* value <0.05 were considered as statistically significant.

**Results:**

The proportion of immediate postpartum anaemia was 24.3%. Frequency of antenatal care (ANC) visits <4 times [AOR = 2.40; 95% CI (1.29, 4.43)], antepartum haemorrhage (APH) [AOR = 5.08; 95% CI (1.91, 13.55)], postpartum haemorrhage (PPH) [AOR = 4.47; 95% CI (2.25, 8.88)], giving birth assisted by instruments (vacuum or forceps) [AOR = 3.99; 95% CI (1.42, 11.23)], poor adherence to iron and folic acid (IFA) [AOR = 2.52; 95% CI (1.06, 6.04)], and midupper arm circumference (MUAC) <23 cm [AOR = 3.25; 95% CI (1.87, 5.65)] were the predictors.

**Conclusion:**

The proportion of immediate postpartum anaemia was a moderate public health concern. ANC, APH, PPH, mode of delivery, adherence to IFA supplementation, and MUAC measurement were the factors affecting the magnitude of anaemia. Therefore, interventions that would address the above mentioned factors need to be implemented.

## 1. Introduction

The postnatal period is a critical phase in the lives of mothers and newborn babies. Even though most maternal and infant deaths occur during this time, this is the most neglected period for the provision of quality of care, especially in low resource setting countries [1].

There is no consensus on the definition of postpartum anaemia. Nevertheless, as it can be inferred from the definition given by different scholars, postpartum anaemia (PPA) occurs when haemoglobin level <11 gm/dl at 1 week and <12 gm/dl at 8 weeks of the postpartum period [2, 3]. Accordingly, haemoglobin level 10–10.9 gm/dl is categorized as mild anaemia, 7–9.9 gm/dl and <7 gm/dl are categorized as moderate and severe anaemia, respectively [4]. Furthermore, even if there is no clear agreement as to the right time to determine the postpartum haemoglobin level, it is usually recommended to check on the first postpartum day [5, 6].

The prevalence of anaemia among postnatal mothers in developed countries ranges from 10% to 30% and in developing countries 50% to 80% [2, 6]. In Ethiopia despite of the 2020 anaemia reduction plan, postpartum anaemia among lactating women increased from 18% in 2011 to 28.6% in 2016 [7, 8]. Anaemia is an indirect cause of maternal morbidity and mortality which accounts for 2% of total maternal mortality in Ethiopia [8, 9]. Blood loss and anaemia were interrelated causes of maternal complications. Due to bleeding, postnatal mothers lose a significant amount of iron during labour and delivery [10, 11]. PPA is also strongly associated with poor quality of life, palpitation, increase maternal infection, fatigue, reduced cognitive ability, emotional instability, and postpartum depression. These outcomes may, in turn, result in poor mother-child bonding, inability to provide care and breastfeeding, or slow infant development [2, 12, 13].

The studies conducted in different parts of the world revealed that factors like young maternal age (<21 years) [14], low educational status of the mother [15], rural residence [16], cesarean mode delivery, episiotomy assisted delivery, prenatal anaemia [17–19], antepartum haemorrhage (APH), postpartum haemorrhage (PPH) [20], antenatal care (ANC) visit less than four [21], reactive (positive) in Human Immunodeficiency Virus/Acquired Immune Deficiency Syndrome (HIV/AIDS) test, malaria [15, 16, 22], and poor adherence of iron and folic acid (IFA) intake during pregnancy [15, 23–25] were some of the independent factors significantly associated with the occurrence of postpartum anaemia. The reduction of postpartum anaemia is a component of target 2 (50% anaemia reduction plan) of World Health Organizations' (WHO) to achieve sustainable development goals.

Some studies were conducted in Ethiopia on anaemia during pregnancy, yet postpartum anaemia screening is the least emphasized illness in the postpartum period. Early diagnosis and identifying the possible risk factors are helpful to manage PPA on time before further complications developed. This study might provide insight into postpartum anaemia to health care providers to propose targeted screening and intervention measures for those whose haemoglobin level <11 gm/dl. Furthermore, researchers might also be benefited by using this result as baseline data to conduct further community-based studies covering a wide area. Several factors contribute to PPA and it can be difficult to generalize the causes for all mothers who reside in a different area. Therefore, this study was intended to assess the proportion and associated factors of anaemia among immediate postnatal mothers in Debre Markos Referral Hospital, Northwest Ethiopia.

## 2. Material and Methods

### 2.1. Study Setting, Design, and Period

This study was conducted in Debre Markos Referral Hospital which is located in East Gojjam Zone, Northwest Ethiopia, and 299 km far from Addis Ababa, the capital city of Ethiopia. This hospital serves more than 3.5 million people who reside in the town and neighbouring areas. In the maternity department, a total of 7 gynaecologists, 1 emergency surgeon, 1 MSc clinical midwife, 14 general practitioners, and 36 midwives work as health care providers. This department has 60 beds for inpatient clients to serve high-risk mothers, gynecologic case-patients, and postnatal mothers. The annual delivery report showed that 6017 mothers gave childbirth in this hospital. The study was conducted by using an institutional-based cross-sectional study design method and data was collected from August 1^st^ to October 30^th^, 2019.

### 2.2. Source and Study Population

All postnatal mothers who gave birth in Debre Markos Referral Hospital and mothers who gave birth somewhere else but came to the hospital within 24 hours of the postpartum period were considered as source population. Mothers who fulfilled the criteria to be a source population and avail themselves during the data collection period were the study population.

### 2.3. Inclusion and Exclusion Criteria

All postnatal mothers who gave birth in Debre Markos Referral Hospital and mothers who gave birth somewhere else but arrived at the hospital within 24 hours during the data collection period were included in this study. Mothers who were anaemic before conception and/or during pregnancy were excluded in this study.

### 2.4. Sample Size Determination

Sample size calculation was based on single population proportion formula by using the following assumption: 50% proportion since there were no the same previous studies, 95% confidence level, and 5% margin of error, and then the calculated sample size was 385. Finally, by adding a 10% nonresponse rate, the final sample size was 424. Sample size determination by using the second objective (statistically significant factors) was calculated by Epi info version 7.2.1, and the maximum sample size was 312. Therefore, the sample calculated by the first objective was larger than the sample size determined by the second objective. Therefore, the final sample size for this study was 424.

### 2.5. Sampling Technique and Procedure

Systematic random sampling was employed to select the study participants. Based on 3 consecutive previous month's average number of delivery data (497), bed numbers in maternity unit (60), and sample size (424); interval was computed. Beds occupied by postnatal mothers were selected with a systematic random sampling technique every 8^th^ bed interval. After written informed consent was taken, data were collected through interviews and chart reviews. Blood samples were collected and processed using the standard procedures for haemoglobin determination at 24 hours before mothers have discharged from the hospital.

### 2.6. Study Variables

#### 2.6.1. Dependent Variable

Dependent variable is immediate postpartum anaemia.

#### 2.6.2. Independent Variables

*Sociodemographic related variables* include age, maternal education level, maternal occupation, religion, residence, marital status, husband education level, husband occupation, and estimated average monthly income. *Obstetrical related variables* include parity, antepartum haemorrhage, multiple pregnancies, abortion, interpregnancy interval, antenatal care visit, frequency of antenatal care visit, gestational age at initial (first) ANC visit, place of delivery, mode of delivery, duration of second-stage labour, episiotomy, perineal tear, the weight of newborn, and postpartum haemorrhage. *Coexisting infections related variables* include helminths infestation, malaria infection, medical disease during pregnancy, HIV/AIDS, syphilis, and urinary tract infection. *Dietary and micronutrient uptake related variables* include hot drink (tea, coffee, or milk) when she has taken iron, meal frequency per day, adherence of IFA, and midupper arm circumference (MUAC).

### 2.7. Operational Definitions

Immediate postpartum period is the first 24 hours after childbirth [26].

Postpartum anaemia is when the haemoglobin level is less than 11 gm/dl at 24 hours of the postpartum period [19, 27].

Adherence of iron and folic acid supplementation means women who had taken iron folate supplements ≥90 days during the most recent pregnancy [8].

### 2.8. Data Collection Tools and Procedures

The questionnaire was developed after reviewing different pieces of literature [20, 21, 28] conducted in different parts of the world. First, the questionnaire was prepared in English and translated into a local language Amharic and then backto English to keep consistency. Two BSc midwives and one master midwife were recruited for data collection and supervision, respectively. After taking written informed consent, data were collected through both face-to-face interview and retrospective maternal chart review by using a semistructured pretested questionnaire. MUAC was measured via tape measures on the nondominant hand, mostly left hand. The result was interpreted to the United Nation International Children's Emergency Fund (UNICEF) and WHO recommendations of cutoff point <23 cm as undernourished and ≥23 cm as well nourished. About 1-2 milliliter of venous blood was collected from each study participant aseptically for haemoglobin estimation. Then, haemoglobin was determined using automated blood analyzer Cell-Dyne 1800 (Abbot Laboratories Diagnostic Division, USA) by experienced laboratory technologist. Finally, the level of haemoglobin was collected and attached to their respective charts. At the end, anaemic mothers were managed with iron and folate or transfused blood based on their haemoglobin levels and advised on iron-rich diets intake.

### 2.9. Data Quality Control

The training was provided for both data collectors and a supervisor. The questionnaire was pretested at Debre Berhan referral hospital with 5% of the sample size. Completeness, clarity, and appropriateness of the questionnaire were modified accordingly after the pretest. Completeness of data was checked daily.

### 2.10. Data Processing and Analysis

Collected data were checked for completeness, consistency, clarity, and missed values. Then, it was cleaned, coded, and entered using Epi Data version 4.6.0.0 and then exported to SPSS (Statistical Package for Social Science) version 24 for analysis. Descriptive analysis: frequency, proportion, mean, median, and standard deviation were computed and presented with texts, tables, and graphs. The normality assumption was checked with a histogram and box plot. Multicollinearity and chi-square assumptions were done. Then, binary logistic regression was performed and variables at *p* value <0.2 were entered into a multivariable logistic regression to control possible confounders. Crude and adjusted odds ratios with 95% confidence interval were computed. Finally, variables at *p* value <0.05 were considered as statistically significant.

## 3. Results

### 3.1. Sociodemographic Characteristics

Four hundred twenty-four immediate postnatal mothers were involved in this study. The mean age of the study population was 27.79 years with a standard deviation of ±5.12. Based on the age category, 241 (56.8%) study participants were with the age range of 25–34 years. More than one-third (35%) of mothers were unable to read and write and two hundred seventy-seven (65.3%) of mothers were housewives by their occupation. Almost all (95.5%) were orthodox Christian religion followers. More than half (55.7%) of the participants were from urban areas. More than one-third (40.9%) of husbands were farmers. Most of the participants' husband might be predominantly involved in farming activities and urban farming is also a common practice these days. Regarding estimated average monthly income, 158 (39.2%) of the respondents' income was within the range of 1000–3000 birr with a median income of 3500 birr and IQR ± 3500 birr. (Table 1).

### 3.2. Obstetrical Related Factors

Among the total 424 study participants, 218 (51.5%) were primipara mothers. Fifty-six (25%) of the mothers had a short interpregnancy interval which was less than two years. A majority (88.4%) of the study participants had antenatal care follow-up during the most recent pregnancy. From those, more than half (60.6%) of the mothers had ≥4 ANC visits, and two hundred fifty-nine (69.1%) of them started the follow-up before 16 weeks of gestation. Of the total study participants, 27 (6.4%) of them had an antepartum haemorrhage in the latest pregnancy. Almost all (95.0%) of the study participants gave birth in health institutions and more than half (52.6%) of the study participants gave childbirth through cesarean section, and 89.5% of the operations were emergency/unplanned. (Table 2).

### 3.3. Coexisting Infection-Related Factors

**Figure 1:**
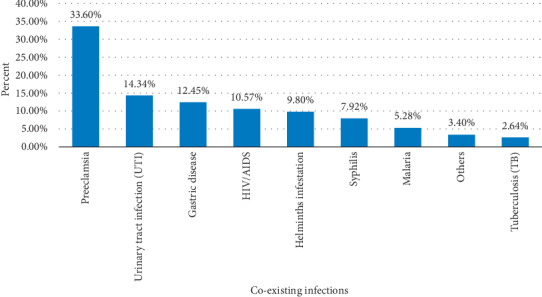
Magnitude of coexisting infections among postnatal mothers in Debre Markos Referral Hospital, Northwest Ethiopia, 2019. ^*∗*^Others include liver disease, heart disease, and pneumonia.

One hundred twelve (26.5%) of the respondents had clinically confirmed medical and parasitic illness before or/and during the latest pregnancy. Among these, preeclampsia was the most frequent complaint (33.60%), whereas tuberculosis was the least one (2.64%). (Figure 1).

### 3.4. Dietary and Micronutrients Utilization Related Factors

Three hundred sixty-eight of the study participants were started on IFA tablets during the most recent pregnancy. Among these, 176 (51.5%) were started before 16 weeks of gestation, whereas 45.9% and 2.6% were started during second- and third-trimester pregnancy, respectively. Among those mothers who took IFA tablets, only 93 (27.3%) of them had good adherence. Among IFA tablets supplied mothers, 135 (39.5%) participants drank hot drink when they took iron. More than two-thirds of 322 (76.1%) of the mothers ate three or fewer times per day during pregnancy. Close to half (46%) of the mothers' midupper arm circumference was less than 23 cm. (Table 3).

### 3.5. Proportion and Associated Factors of Immediate Postpartum Anaemia

#### 3.5.1. The Proportion of Immediate Postpartum Anaemia

**Figure 2:**
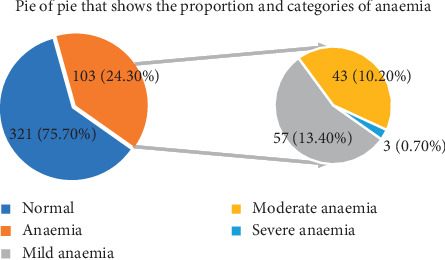
Categories of immediate postpartum anaemia among postnatal mothers in Debre Markos Referral Hospital, Northwest Ethiopia, 2019 (*n* = 424).

Postpartum anaemia was observed among 103 (24.3%) mothers. Postpartum haemoglobin concentrations of study participants ranged from 6.70 gm/dl to 17.50 gm/dl with a mean value of 12.4 gm/dl and SD ± 1.88 gm/dl. From the total 24.3% anaemic mothers, 13.4%, 10.2%, and 0.7% of them were categorized as mild, moderate, and severe anaemia, respectively (Figure 2).

#### 3.5.2. Associated Factors of Postpartum Anaemia

In binary logistic regression analysis, maternal educational level (being unable to read and write and able to read and write), rural residence, home delivery, preterm delivery, and less frequent meal per day were variables associated with immediate PPA with *p* value <0.2, but all these variables were eliminated by backward multivariate regression. In multivariable logistic regression, antenatal care visit <4 times, antepartum haemorrhage, instrumental delivery, postpartum haemorrhage, poor adherence to IFA supplementation, and MUAC <23 cm were independent variables significantly associated with *p* value <0.05.

The odds of postpartum anaemia were higher among postnatal women who had <4 antenatal care visits [AOR = 2.40; 95% CI (1.29, 4.43)]. The higher likelihood of postpartum anaemia was observed among postnatal women who were experienced APH and PPH compared to their counterparts [AOR = 5.08; 95% CI (1.91, 13.55)] and [AOR = 4.47; 95% CI (2.25, 8.88)], respectively. Mothers who gave birth by instrumental assisted vaginal delivery were almost 4 times more likely to be anaemic compared to those who gave birth through spontaneous vaginal delivery [AOR = 3.99; 95% CI (1.42, 11.23)]. Mothers who poorly adhere to IFA supplementation were 2.5 times more likely to develop postpartum anaemia compared to those who had good adherence [AOR = 2.52; 95% CI (1.06, 6.04)]. Increased odds of anaemia were noted among postpartum women with MUAC measurements of <23 cm [AOR = 3.25; 95% CI (1.87, 5.65)] (Table 4).

## 4. Discussion

This study assessed the proportion and factors associated with immediate postpartum anaemia among postnatal mothers at 24-hour postpartum period in Debre Markos Referral Hospital. The proportion of immediate postpartum anaemia was 24.3%.

This value was in line with the study done in Germany (22%), Jimma (28.7%), Costal Karnataka (26.5%), and Mekelle (24.2%), respectively [20, 21, 24, 29]. But, haemoglobin cutoff points to define anaemia and the time (postpartum period) to diagnose anaemia were different.

The magnitude of immediate postpartum anaemia in this study was lower than the study done in Uganda (30%) [17]; Madrid, Spain (29%) [30]; Mancha Centro hospital, Spain (45%) [28]; Tamil Nadu, India (47.3%) [23]; Pakistan (47.9%) [15]; and Myanmar 73.8% [27]. The possible reason for this variation might be due to anaemic mothers in preconception and pregnancy period being excluded from this study, use of different cutoff points to define postpartum anaemia, and difference in postpartum time of screening. Due to a lack of consensus on the definition of PPA, different scholars use different cutoff points, like Hgb<12 mg/dl at 10 weeks in Uganda, Hgb <11 mg/dl at 24 hours postpartum in Mancha Centro hospital, Spain, and Hgb <12 mg/dl among all lactating in Myanmar. Geographical difference might be also another factor for the abovementioned variation. Additionally, a staple food in Asian countries is rice, which lacks iron content. Ethiopians are eating cereals, “teff injera” (ferment teff flour), and fruits that do have high iron content [21].

However, this proportion was higher than the Amhara region DHS data report (17.2%) [6]; the study was done in Kenya (16.4%) [8] and Ghana (16%) [31]. The variations might be because the above studies cover a wide range of areas and include all breastfeeding mothers who were far from the immediate postpartum period or close to 6-week and 6-month postpartum period. This shows that when the time of the postpartum period extends, mothers will have enough time to recover from anaemia or haemoglobin level will be increased [32].

Independent factors significantly associated with immediate postpartum anaemia were frequency of ANC visits <4 times in the most recent pregnancy, having antepartum haemorrhage during most recent pregnancy, instrumental assisted vaginal delivery, having the experience of primary postpartum haemorrhage during the most recent birth, poor adherence to IFA supplementation during the latest pregnancy, and MUAC <23 cm at the time of interview.

A majority (88.4%) of the study participants had ANC follow-up and 39% of them had <4 times of visits. The odds of postnatal mothers who had <4 times of antenatal care visits were about 2.4-fold higher to develop postpartum anaemia than those who had ≥4 ANC visits. This finding was supported by the studies conducted in Jimma, Tigray, and Ethiopia DHS data [21, 33, 34]. The possible explanations are as follows: mothers who had <4 ANC visits might not get enough IFA supplements, have poor adherence to IFA supplementation, and not screened/detected the risk factors early and treat on time.

Among all study participants, 27 (6.4%) of them had an antepartum haemorrhage. Mothers who had antepartum haemorrhage were 5 times more likely to be anaemic in the immediate postpartum period, compared to their counterparts. This finding was consistent with the finding from Germany [20]. The possible explanation might be due to the loss of iron stores during pregnancy and blood loss during delivery could be the complications of APH.

The odds of anaemia among postpartum mothers who experienced massive postpartum blood loss were 4.5 times higher than the odds of anaemia among postnatal mothers who did not develop postpartum haemorrhage. The similar findings were reported in Germany, Saudi Arabia, and Tamil Nadu, India [14, 20, 35]. Excessive bleeding after birth decreases the red blood cell component called haemoglobin. In every milliliter blood loss, a half milligram of iron will be reduced in the blood [11].

Mothers who gave birth by instrumental (vacuum or forceps) assisted mode of delivery were almost 4 times more likely to be anaemic in the postpartum period when compared to those who gave birth through spontaneous vaginal delivery. This finding agreed with the studies done in Spain (two studies) and Saudi Arabia [11, 18, 35]. It might be due to the fact that instrumental assisted vaginal delivery increases the risk of episiotomy, spontaneous perineal, or/and cervical tear, and this tear may be also extended to the uterus. Clinicians are usually misdiagnosing the tears and repairing after mothers bleed a lot.

The odds of PPA were 2.5-fold higher among postpartum mothers who had poor adherence to IFA supplementation compared to their counterparts. This finding was in agreement with the studies carried out in Uganda, Pakistan, and Tanzania [15, 23, 25]. The possible explanation might be due to the depleting of stored maternal iron since physiologic requirements of iron during pregnancy and labour are high. Therefore, not taking IFA based on the right order could reduce iron store and results in anaemia even with minimal blood loss during childbirth.

Increased odds of anaemia were noted among postnatal mothers whose MUAC measurements <23 cm compared to those whose MUAC measurements ≥23 cm. This study is supported by the study done in Jimma, Myanmar, and Tanzania [21, 25, 36]. The most likely explanation might be iron deficiency anaemia usually related to nutritional deficiency. MUAC measurement <23 cm indicates that poor muscle mass lacks adequate energy intake. Haemoglobin concentration and maternal MUAC had a linear relationship which was also another explanation [16].

### 4.1. Limitation of the Study

Since the interview was about the past nine months' activity of mothers, recall bias was one of the limitations. Besides, environmental and behavioural factors were not assessed. The study was facility based and difficult to generalize for the entire community. As the study design was a cross-sectional, a causal relationship could not be established.

## 5. Conclusions

The proportion of postpartum anaemia was a moderate public health problem in Debre Markos Referral Hospital. Antepartum haemorrhage, instrumental assisted vaginal delivery, frequency of antenatal care visit <4 times, postpartum haemorrhage, poor adherence to IFA supplementation, and MUAC less than 23 cm were independent factors significantly associated with immediate postpartum anaemia. Therefore, interventions that would address the abovementioned factors need to be implemented, and further researches that will address this study limitation should be also considered.

## Figures and Tables

**Table 1:** Sociodemographic related characteristics of postnatal mothers in Debre Markos Referral Hospital, Northwest Ethiopia, 2019 (*n* = 424).

Variables	Category	Frequency	Percent (%)
Age	15–24	121	28.6
	25–34	241	56.8
	35–49	62	14.6

Maternal education status	Unable to read and write	148	35.0
	Able to read and write	49	11.6
	Primary class completed	55	13.0
	Secondary class completed	81	19.1
	Diploma and above	90	21.3

Occupation of respondent	Housewife	277	65.3
	Government employee	73	17.2
	Private employee	25	5.9
	Merchant	38	9
	Others^*∗*^	11	2.6

Religion	Orthodox	405	95.5
	Muslim	14	3.3
	Protestant	5	1.2

Residence	Rural	188	44.3
	Urban	236	55.7

Marital status	Married	408	96.2
	Unmarried	16	3.8

Husband education level	Unable to read and write	133	32.3
	Able to read and write	31	7.5
	Primary class completed	55	13.4
	Secondary class completed	80	19.4
	Diploma and above	113	27.4

Husband occupation	Government employee	104	25.5
	Private employee	68	16.7
	Merchant	49	12
	Farmer	167	40.9
	Others^*∗*^	20	4.9

Monthly income (ET birr)	<1000	18	4.5
	1000–3000	158	39.2
	3001–5000	102	25.3
	≥5001	125	31.0

^*∗*^Others represent daily labourers and students for both occupation of study participants (mothers) and husbands.

**Table 2:** Obstetrical related characteristics of postnatal mothers in Debre Markos Referral Hospital, Northwest Ethiopia, 2019 (*n* = 424).

Variables	Category	Frequency	Percent (%)
Parity	Primipara	218	51.5
	Para 2–4	171	40.2
	Grand multiparous	35	8.3

History of abortion	Yes	71	31.6
	No	154	68.4

Interpregnancy interval in years	<2	56	25.0
	≥2	168	75.0

Attend ANC during this pregnancy	Yes	375	88.4
	No	49	11.6

GA when ANC visit initiated in weeks	<16	259	69.1
	16–24	69	18.4
	25–30	45	12.0
	31–34	2	0.5

Frequency of ANC visits (times)	<4	148	39.4
	≥4	227	60.6

Antepartum haemorrhage	Yes	27	6.4
	No	397	93.6

Multiple pregnancies	Yes	23	5.4
	No	401	94.6

Place of birth	Health institution	403	95.0
	Home	21	5.0

Mode of delivery	SVD	176	41.5
	IAVD	25	5.9
	C/S	223	52.6

Type of C/S	Elective	25	10.5
	Emergency	198	89.5

Manual removal of placenta	Yes	20	4.7
	No	404	95.3

Episiotomy	Yes	19	4.5
	No	405	95.5

Prolonged second stage	Yes	32	7.5
	No	392	92.5

Perineal tear	Yes	41	9.7
	No	383	90.3

Weight of newborn in gram	<2500	72	17.0
	2500–3999	348	82.1
	≥4000	4	0.9

Postpartum haemorrhage	Yes	60	14.2
	No	364	85.8

ANC: antenatal care, GA: gestational age, IAVD: instrumental assisted vaginal delivery, SVD: spontaneous vaginal delivery, and C/S: cesarean section.

**Table 3:** Dietary and micronutrient uptake characteristics of postnatal mothers in Debre Markos Referral Hospital, Northwest Ethiopia, 2019 (*n* = 424).

Variables	Category	Frequency	Percent (%)
IFA tablet took during pregnancy	Yes	368	86.8
	No	56	13.2

GA when IFA tablet started	<16 weeks	176	51.5
	20–24 weeks	90	26.3
	26–30 weeks	67	19.6
	30–34 weeks	9	2.6

Adherence to iron and folic acid supplementation	Poor adherence	248	72.7
	Good adherence	93	27.3

Hot drink while taking iron	Yes	135	39.5
	No	207	60.5

Frequency of meal per day	≤3	322	76.1
	>3	101	23.9

Midupper arm circumference in centimeter (cm)	<23	195	46.0
	≥23	229	54.0

IFA: iron and folic acid, GA: gestational age.

**Table 4:** Logistic regression showing factors associated with immediate postpartum anaemia among postnatal mothers in Debre Markos Referral Hospital, Northwest Ethiopia, 2019 (*n* = 424).

Independent variables	Postpartum anaemia		COR 95% CI	AOR 95%CI
	Yes	No		
Maternal education status				
Unable to read and write	55	93	5.32 (2.48–11.44)	2.00 (0.81–4.88)
Able to read and write	14	35	3.60 (1.43–9.09)	2.05 (0.71–5.87)
Primary school class	10	45	2.00 (0.76–5.23)	1.66 (0.57–4.80)
Secondary school class	15	66	2.04 (0.85–4.98)	1.09 (0.40–2.99)
Diploma and above	9	81	1	1

Residence				
Rural	59	129	2.00 (1.27–3.13)	0.63 (0.31–1.29)
Urban	44	192	1	1

Frequency of ANC visits				
<4 times	46	102	3.10 (1.84–5.25)	2.40 (1.29–4.43)^*∗*^
≥4 times	29	198	1	1

Antepartum haemorrhage				
Yes	17	10	6.15 (2.71–13.9)	5.08 (1.91–13.55)^*∗*^
No	86	311	1	1

GA at delivery				
Preterm	31	58	1.96 (1.18–3.25)	1.41 (0.73–2.71)
Term	69	255	1	1

Place of birth				
Health institution	92	311	1	1
Home	11	10	3.72 (1.61–9.00)	1.92 (0.60–6.19)

Mode of delivery				
SVD	39	137	1	1
IAVD	13	12	3.81 (1.57–8.77)	3.99 (1.42–11.23)^*∗*^
Cesarean section	51	172	1.04 (0.65–1.67)	1.06 (0.59–1.88)

Postpartum haemorrhage				
Yes	36	24	6.65 (3.72–11.89)	4.47 (2.25–8.88)^*∗*^
No	67	297	1	1

Adherence of IFA supplementation				
Poor adherence	53	195	2.88 (1.31–6.33)	2.52 (1.06–6.04)^*∗*^
Good adherence	8	85	1	1

Frequency of meal per day				
≤3	87	235	1.97 (1.09–3.54)	1.21 (0.59–2.48)
>3	16	85	1	1

Midupper arm circumference				
<23 cm	71	124	3.53 (2.12–5.66)	3.25 (1.87–5.65)^*∗*^
≥23 cm	32	197	1	1

GA: gestational age; SVD: spontaneous vaginal delivery; IAVD: instrumental assisted vaginal delivery; IFA: iron and folic acid; AOR: adjusted odds ratio; COR: crude odds ratio; 1: reference and ^*∗*^significantly associated with *p* value <0.05.

## Data Availability

The datasets used and/or analyzed during the current study are available from the corresponding author upon reasonable request.

## Abbreviations

ANC: Antenatal care
AOR: Adjusted odds ratio
APH: Antepartum haemorrhage
COR: Cruds odds ratio
CS: Cesarean section
DHS: Demographic health survey
GA: Gestation age
HIV: Human immune virus
IAVD: Instrumental assisted vaginal delivery
IFA: Iron and folic acid
MUAC: Midupper arm circumference
OR: Odds ratio
PPA: Postpartum anaemia
PPH: Postpartum haemorrhage
SPSS: Statistical package for social science
SVD: Spontaneous vaginal delivery
TB: Tuberculosis
VDRL: Venereal disease research history
WHO: World Health Organization.
